# Bristol Final FRCS Course

**Published:** 1983-10

**Authors:** D. J. Leaper


### Bristol Final FRCS Course

Sir

The Final FRCS examination is said to be easy to fail but not difficult to pass. The two-week course held annually in Bristol over the last 6 years is designed primarily to aid candidates gain experience of examination conditions which they will meet when they take the examination at one of the Royal Colleges of Surgeons. Poor viva voce technique is one of the largest hurdles on the day and has failed many an able candidate. This year we have swung even further away from formal lectures and demonstrations and concentrated on this aspect.

Nevertheless a large portion of the course is taken up by traditional teaching which this year was given by 18 external teachers; 13 of whom were from outside the South West Region including 4 professors. Our internal teachers numbered 46 consultants and 23 junior staff. All aspects of the principles and practice of surgery, operative surgery, surgical pathology and radiology, and most important, clinical cases were covered in general surgery (with emphasis on gastroenterology and vascular surgery), urological, orthopaedic, plastic, paediatric, thoracic and neurosurgery.

Twenty-four candidates attended the course this year, half of them were from the South West Region. Judging by their response this year's course was certainly successful. We invite the candidates to mark each session and with very few exceptions the teachers achieved perfect scores! The orthopaedic clinical cases session deserves particular mention in this respect.

So how will our candidates fare in the real thing? Of last year's 25 registrants, 17 (68%) are known to have passed the Final Fellowship examination at one, at least, of the Royal Colleges of Surgeons within 9 months of the Bristol course: 5 are known to have failed and 3 cannot be traced.

D. J. Leaper

